# Chronic Kidney Disease Progression Risk in Patients With Diabetes Mellitus Using Dihydropyridine Calcium Channel Blockers: A Nationwide, Population-Based, Propensity Score Matching Cohort Study

**DOI:** 10.3389/fphar.2022.786203

**Published:** 2022-03-09

**Authors:** Shih-Yi Lin, Cheng-Li Lin, Cheng-Chieh Lin, Wu-Huei Hsu, Chung-Y. Hsu, Chia-Hung Kao

**Affiliations:** ^1^ Graduate Institute of Biomedical Sciences, College of Medicine, China Medical University, Taichung, Taiwan; ^2^ Division of Nephrology and Kidney Institute, China Medical University Hospital, Taichung, Taiwan; ^3^ Management Office for Health Data, China Medical University Hospital, Taichung, Taiwan; ^4^ College of Medicine, China Medical University, Taichung, Taiwan; ^5^ Department of Family Medicine, China Medical University Hospital, Taichung, Taiwan; ^6^ Department of Chest Medicine, China Medical University Hospital, Taichung, Taiwan; ^7^ Department of Nuclear Medicine, PET Center, China Medical University Hospital, Taichung, Taiwan; ^8^ Department of Bioinformatics and Medical Engineering, Asia University, Taichung, Taiwan; ^9^ Center of Augmented Intelligence in Healthcare, China Medical University Hospital, Taichung, Taiwan

**Keywords:** NHIRD: national health insurance, diabetes mellitus (DM), chronic kidney disease (CKD), dihydropyridine calcium channel blockers (DCCBs), end-stage renal disease (ESRD)

## Abstract

**Background:** Whether diabetes mellitus (DM) patients with chronic kidney disease (CKD) can glean individual renal benefit from dihydropyridine calcium channel blockers (DCCBs) remains to be determined. We conducted a nationwide, population-based, propensity score matching cohort study to examine the effect of DCCBs on CKD progression in DM patients with CKD.

**Methods:** One million individuals were randomly sampled from Taiwan’s National Health Insurance Research Database. The study cohort consisted of DM patients with CKD who used DCCBs. The comparison cohort was propensity-matched for demographic characteristics and comorbidities. The endpoint was advanced CKD or end-stage renal disease (ESRD). The Cox proportional hazards model was used to calculate the risks.

**Results:** In total, 9,761 DCCB users were compared with DCCB nonusers at a ratio of 1:1. DCCB users had lower risk of advanced CKD and ESRD than nonusers—with adjusted hazard ratio [aHR; 95% confidence interval (CI)] of 0.64 (0.53–0.78) and 0.59 (95% CI, 0.50–0.71) for advanced CKD and ESRD, respectively. DCCB users aged ≥65 years had the lowest incidence rates of advanced CKD and ESRD—with aHR (95% CI) of 0.47 (0.34–0.65) and 0.48 (0.35–0.65) for advanced CKD and ESRD, respectively. Finally, cumulative DCCB use for >1,100 days was associated with the lowest advanced CKD and ESRD risks [(aHR, 0.29 (95% CI, 0.19–0.44)].

**Conclusion:** DM patients with CKD who used DCCBs had lower risk of progression to advanced CKD and ESRD than nonusers did.

## Introduction

Dihydropyridine calcium channel blockers (DCCBs), which bind to calcium channels located on vascular smooth muscles to interrupt calcium entry, are widely used in clinical practice to reduce blood pressure (Braunwald, 1982; Eisenberg et al., 2004). In addition, DCCBs can provide many cardiovascular (CV) benefits (Staessen et al., 1997; Nissen et al., 2004; Hasebe and Kikuchi, 2005; Haller, 2008). A meta-analysis of 175,634 patients demonstrated that DCCBs could reduce all-cause mortality and stroke risk and prevent heart failure (Costanzo et al., 2009). Systematic reviews have shown that DCCBs could reduce the risk of Parkinson disease (Lang et al., 2015), incidental dementia (Hussain et al., 2018), and sequelae of traumatic brain injury (Gurkoff et al., 2017). However, because of their vasodilatory effect, DCCBs have also been reported in association with increased gastrointestinal tract bleeding (Kaplan et al., 2000) and proischemic complication (Waters, 1991) risks.

Currently, whether DCCBs have individual benefits or harms on kidney function, particularly in diabetes mellitus (DM) patients with chronic kidney disease (CKD), who are at the highest risk for end-stage renal disease (ESRD), remains unclear (de Leeuw et al., 2004). This is partly because CKD and CV events share multiple risk factors and medications (Cai et al., 2013) and the other part due to intrinsic characteristic of DCCBs of vasodilatation of afferent arterioles and increased intraglomerular pressure (Carmines and Navar, 1989; Kimura et al., 1994). Although numerous randomized controlled trials have investigated the effects of DCCBs on renal function, most have compared angiotensin-converting enzyme inhibitors (ACEIs)/angiotensin II receptor blockers (ARBs) with DCCBs (Chan et al., 1992; Bakris et al., 1996; Velussi et al., 1996; Campo et al., 1997; Estacio et al., 1998; Lewis et al., 2001; Rahman et al., 2012), combined DCCB–ACEI/ARB therapy with noncombination therapy (Hasebe and Kikuchi, 2005), DCCBs with diuretics (de Leeuw et al., 2004), or combined ACEIs/ARB–DCCB therapy with combined ACEI/ARB–diuretics therapy (Kaneshiro et al., 2009; Kohlmann et al., 2009; Bakris et al., 2010; Doi et al., 2010; Ishimitsu et al., 2011; Lee et al., 2012; Ogihara et al., 2014; Oshikawa et al., 2014). However, most of the aforementioned studies had a short follow-up duration (Campo et al., 1997; Doi et al., 2010; Ishimitsu et al., 2011; Lee et al., 2012), small sample size (Chan et al., 1992; Bakris et al., 1996; Campo et al., 1997; Doi et al., 2010; Ishimitsu et al., 2011), intrinsic heterogeneous population (CKD, DM, diabetic CKD, or diabetic non-CKD) (Fernández et al., 2001; Bakris et al., 2010; Ishimitsu et al., 2011), or did not consider possible effects of hypoglycemic agents on renal function in patients with DM (Chan et al., 1992; Bakris et al., 1996; Velussi et al., 1996; Fernández et al., 2001). Whether DCCBs have long-term “individual” effects on renal function remain to be determined. In their study involving patients with type 2 DM, Bakis et al. reported comparable effects of DCCBs and ACEIs in slowing renal disease progression; however, the sample size was only 52 patients (Bakris et al., 1996). We hypothesized that DCCBs have individual effects on delaying CKD progression, particularly in DM patients with CKD. We conducted a nationwide, population-based, propensity score matching study to examine the effects of DCCBs on CKD progression in DM patients with CKD.

## Methods

### Data Source

In 1995, Taiwan launched a single-payer healthcare system from which the National Health Insurance (NHI) Research Database (NHIRD) was established. Data used in the present study were obtained from the Longitudinal Health Insurance Database, which contains information on 1 million individuals randomly sampled from NHIRD. The data were deidentified to protect privacy. The medical claims data contained information on diagnosis, outpatient visits, hospital admissions, drug prescriptions, and surgical procedures. The diagnostic classification was based on the International Classification of Diseases, Ninth Revision, Clinical Modification (ICD-9-CM). The study was approved by the Research Ethics Committee at China Medical University and Hospital [CMUH104-REC2-115 (AR-4)].

### Study Population

We enrolled DM patients who had subsequent new diagnosis of CKD (ICD-9 codes 585.xx). In this study, those who had the record of DM (ICD-9 codes 250.x0 or 250.x2) more than two times within 1 year were defined as DM patients. CKD was defined on the basis of ICD-9 codes 585.xx recorded in NHIRD for more than three times. The date of initial DCCB use was defined as the index date. Patients were followed up until date of first erythropoietin (EPO) prescription, onset of ESRD, withdrawal, death, or December 31, 2013. Diagnosis of ESRD was confirmed by ICD-9-CM codes and inclusion in the Registry for Catastrophic Illness Patient Database, a sub-classification of the NHIRD. Although NHIRD lacks information on CKD staging, the NHI program has stated that patients with estimated glomerular filtration rate (eGFR) < 15 ml/min/1.73 m^2^ and hemoglobin level <10 gm/dL may receive the official EPO prescription benefit package. Thus, in the present study, we defined patients with advanced CKD as those with CKD who started EPO prescriptions.

We excluded patients diagnosed as having advanced CKD or ESRD before the index date. A total of 9,761 DCCB users were compared with DCCB nonusers at a ratio of 1:1 using propensity scores after matching for demographic characteristics and baseline comorbidities. Baseline comorbidities included cancer, hyperlipidemia, stroke, chronic obstructive pulmonary disease, cirrhosis, arrhythmia, congestive heart failure, fibromyalgia, coronary artery disease, alcohol-related diseases, peripheral arterial occlusive disease, renal stone, and peptic ulcer disease (list of codes were presented in Supplementary Table S1). The covariates of baseline medications comprised statins, ACEIs/ARBs, loop diuretics, thiazides, potassium-sparing diuretics, non-DCCBs, alpha-blockers, beta-blockers, insulins, sulfonylureas, biguanides, miglitol, acarbose, thiazolidinedione, dipeptidyl peptidase-4 inhibitors, and other antidiabetics.

### Statistical Analysis

Descriptive analyses of demographics, comorbidities, and medications of the cohorts are presented as frequencies and percentages for the categorical variables and as means and standard deviations for the continuous variables. The 1:N Case-Control Matching Macro (Parsons LS et al. SUGI 29) was used to match cases with controls (Parsons, 2012). DM patients with CKD who used DCCB were matched (1:1 ratio) with those who did not use DCCB according to their propensity score through nearest neighbor matching, initially to the eighth digit and then as required to the first digit. Therefore, matches were first made within a caliper width of 0.0000001, and then the caliper width was increased for unmatched cases to 0.1. We reconsidered the matching criteria and performed a rematch (greedy algorithm). For each DM patient on DCCB use, the corresponding comparisons were selected based on the nearest propensity score. A standardized mean difference of ≤0.1 indicates a negligible difference between the two cohorts. Absolute standardized mean difference per covariate before and after PSM was presented in Table 1 and Supplementary Table S2. Cumulative incidence rates of advanced CKD and ESRD were calculated based on the Kaplan-Meier method, and the between-cohort comparisons of the cumulative incidence curves were assessed by log-rank tests.

**TABLE 1:** Demographic characteristics and comorbidities in the propensity-score-matched cohorts with and without dihydropyridine Calcium Channel Blocker used among diabetes mellitus patients with chronic kidney disease.

Variable	Dihydropyridine calcium channel blocker		Standard mean difference^a^
	No*N* = 9,761	Yes*N* = 9,761	
	*n* (%)	*n* (%)	
Age, year			
≤49	2,537 (26.0)	2,549 (26.1)	0.003
50–64	4,020 (41.2)	4,070 (41.7)	0.01
65+	3,204 (32.8)	3,142 (32.2)	0.01
Mean ±(SD)^†^	58.5 (13.3)	58.4 (12.6)	0.006
Sex			
Female	4,348 (44.5)	4,380 (44.9)	0.007
Male	5,413 (55.5)	5,381 (55.1)	0.007
Mean aDCSI score (SD)^†^	0.91 (1.48)	0.93 (1.50)	0.009
Duration of diabetes			
Comorbidity			
Cancer	1,485 (15.2)	1,521 (15.6)	0.01
Hyperlipidemia	6,377 (65.3)	6,422 (65.8)	0.01
Stroke	2,179 (22.3)	2,338 (24.0)	0.004
COPD	2,310 (23.7)	2,293 (23.5)	0.004
Cirrhosis	3,677 (37.7)	3,688 (37.8)	0.002
Arrhythmia	1,373 (14.1)	1,382 (14.2)	0.003
Congestive heart failure	1,405 (14.4)	1,453 (14.9)	0.01
Fibromyalgia	2,898 (29.7)	3,045 (31.2)	0.004
Coronary artery disease	3,657 (37.5)	3,777 (38.7)	0.03
Alcohol-related diseases	1,130 (11.6)	1,167 (12.0)	0.01
PAOD	879 (9.01)	879 (9.01)	0.000
Renal stone	1,286 (13.2)	1,252 (12.8)	0.01
PUD	4,440 (45.5)	4,459 (45.7)	0.004
Medication			
Statin	5,840 (59.8)	5,988 (61.4)	0.004
ACEI or ARB	7,600 (77.9)	8,245 (84.5)	0.004
Loop diuretics	5,901 (60.5)	5,935 (60.8)	0.007
Thiazides	6,207 (63.6)	6,293 (64.5)	0.02
Potassium sparing diuretics	3,098 (31.7)	3,120 (32.0)	0.005
Non-Dihydropyridine Calcium Channel Blockers	3,448 (35.3)	3,499 (35.9)	0.01
Alpha-blockers	3,026 (31.0)	3,032 (31.1)	0.001
Beta-blockers	6,847 (70.2)	6,930 (71.0)	0.02
Insulins	6,965 (71.4)	6,970 (71.4)	0.001
Sulfonylureas	8,707 (89.2)	8,744 (89.6)	0.01
Biguanides	8,898 (91.2)	8,935 (91.5)	0.01
Miglitol	220 (2.25)	228 (2.34)	0.005
Acarbose	3,562 (36.5)	3,616 (37.1)	0.01
Thiazolidinedione	3,333 (34.2)	3,406 (34.9)	0.02
Dipeptidyl peptidase-4	8,757 (89.7)	8,788 (90.0)	0.01
Other antidiabetic	5,024 (51.5)	5,091 (52.2)	0.01

a A standardized mean difference of ≤0.1 indicates a negligible difference between the two cohorts. †, t test.

CAD: coronary artery disease.

COPD: chronic obstructive pulmonary disease.

To consider the effect of the frequency variation in the use of DCCBs, DCCB use was considered as a time-dependent covariate in the Cox proportional hazards model. Hazard ratios (HRs) and the corresponding 95% confidence intervals (CIs) for advanced CKD and ESRD were estimated using the Cox proportional hazards models. A two‐tailed *p* value <0.05 was considered statistically significant. The data analysis was generated using SAS (version 9.4) of the SAS System for [Unix] (SAS Institute Inc., Cary, NC, United States), and the figures were created using R.

## Results

Table 1 lists the demographic characteristics and comorbidities in the propensity score–matched cohorts. The average age of DCCB nonusers and DCCB users was 58.5 ± 13.3 and 58.4 ± 12.6 years, respectively; 44.5 and 44.9% of the DCCB nonusers and users were women, respectively. In the profiles of baseline comorbidities and medications, they did not significantly differ between the two cohorts according to standardized mean differences after PS matching in Table 1.

Table 2 presents the incidence values and HRs for advanced CKD or ESRD in the cohorts from Cox proportional hazards models with time-dependent exposure covariates. The incidence rates of advanced CKD and ESRD in DCCB nonusers were 3.27 and 3.64 per 1,000 person-years, respectively. The incidence rates of advanced CKD and ESRD in DCCB users were 2.28 and 2.59 per 1,000 person-year, respectively. The DCCB users had lower advanced CKD and ESRD risk than did nonusers [with HR (95% CI) of 0.71 (0.59–0.85) for advanced CKD and 0.72 (0.61–0.86) for ESRD].

**TABLE 2:** Incidence and HRs of advanced CKD or ESRD in the dihydropyridine calcium channel blocker (DCCB) cohorts compared with those in the non-DCCB cohorts among diabetes mellitus patients with chronic kidney disease by Cox proportional hazard models with time-dependent exposure covariates.

	DCCB	
	No(*N* = 9,761)	Yes(*N* = 9,761)
Advanced CKD		
Person-years	82,063	86,035
Follow-up time (y), Median±(IQR)	905 (5.30–12.0)	9.31 (5.95–12.1)
Event	268	196
Rate^#^	3.27	2.28
HR (95% CI)	1 (Reference)	0.71 (0.59, 0.85)***
ESRD		
Person-years	82,176	86,121
Follow-up time (y), Median±(IQR)	9.05 (5.30–12.0)	9.33 (5.96–12.1)
Event	229	223
Rate^#^	3.64	2.59
HR (95% CI)	1 (Reference)	0.72 (0.61, 0.86)***

Rate#, incidence rate, per 1,000 person-years; HR, relative hazard ratio.

*p < 0.05; **p < 0.01; ***p < .001.

Table 3 lists the incidence and HRs of advanced CKD or ESRD in the cohorts by age and sex from Cox proportional hazards models with time-dependent exposure covariates. Reduced incidence rates of advanced CKD and ESRD in DCCB users were observed in the ≥65-year age group compared with those in DCCB nonusers [with HR (95% CI) of 0.49 (0.36–0.68) in the ≥65-year age group for advanced CKD, respectively, and 0.76 (0.59–1.00) and 0.50 (0.38–0.67) in the 50–64- and ≥65-year age group for ESRD, respectively). Women using DCCBs were at lower advanced CKD and ESRD risks compared with women not using DCCBs [with HR (95% CI) of 0.67 (0.51–0.88) and 0.61 (0.47–0.78) for advanced CKD and ESRD, respectively].

**TABLE 3:** Incidence and hazards ratio of advanced CKD or ESRD measured by age, sex, and comorbidity in the dihydropyridine calcium channel blocker (DCCB) cohorts among diabetes mellitus patients with chronic kidney disease compared with those in the non-DCCB cohorts by Cox proportional hazard models with time-dependent exposure covariates.

Variables	DCCB				HR (95% CI)
	No (*N* = 9,761)		Yes (*N* = 9,761)		
	Event	Rate^#^	Event	Rate^#^	
Advanced CKD					
Age, years					
≤49	42	1.83	49	2.08	1.14 (0.76, 1.73)
50-64	112	3.16	88	2.38	0.76 (0.57, 1.00)
65+	114	4.82	59	2.32	0.49 (0.36, 0.68)***
Sex					
Female	125	3.26	87	2.13	0.67 (0.51, 0.88)**
Male	143	3.27	109	2.41	0.74 (0.58, 0.95)*
ESRD					
Age, years					
≤49	45	1.96	56	2.37	1.22 (0.83, 1.81)
50–64	125	3.52	99	2.67	0.76 (0.59, 1.00)*
65+	129	5.44	68	2.67	0.50 (0.38, 0.67)***
Sex					
Female	155	4.04	98	2.40	0.61 (0.47, 0.78)***
Male	144	3.29	125	2.76	0.85 (0.67, 1.08)

Rate#, incidence rate, per 1,000 person-years; HR, relative hazard ratio.

*p < 0.05; **p < 0.01; ***p < .001.

Table 4 shows incidence values and HRs for advanced CKD or ESRD stratified by duration of DCCB therapy. Compared with nonuse, cumulative DCCB use for >300 days reduced CKD risk (with HR [95% CI] of 0.63 [0.46–0.87] and 0.32 [0.21–0.48] for cumulative DCCB use for 301–1,100 and >1,100 days, respectively). Compared with nonuse, cumulative DCCB use for >300 days reduced ESRD risk [with aHR (95% CI) of 0.66 (0.49–0.89), and 0.28 (0.19–0.42) for cumulative DCCB use for 301–1,100, and >1,100 days, respectively].

**TABLE 4:** Incidence and adjusted hazard ratio of advanced CKD or ESRD stratified by duration of dihydropyridine calcium channel blocker (DCCB) therapy among diabetes mellitus patients with chronic kidney disease.

Medication exposed	*N*	Event	Person-year	Rate	HR (95%CI)
Advanced CKD					
Non- DCCB	9,761	268	82,062	3.27	1.00
DCCB					
≤30 days	2,555	59	19,439	3.04	0.88 (0.66, 1.16)
31-300 days	2,294	69	18,499	3.73	1.10 (0.85, 1.44)
301-1,100 days	2,372	43	21,071	2.04	0.63 (0.46, 0.87)**
>1,100 days	2,540	25	27,025	0.93	0.32 (0.21, 0.48)***
ESRD					
Non-DCCB	9,761	299	82,175	3.64	1.00
DCCB^a^					
≤30 days	2,555	74	19,455	3.80	0.99 (0.77, 1.28)
31-300 days	2,294	74	18,527	3.99	1.07 (0.83, 1.37)
301-1,100 days	2,372	50	21,093	2.37	0.66 (0.49, 0.89)**
>1,100 days	2,540	25	27,045	0.92	0.28 (0.19, 0.42)***

a The cumulative use day are partitioned in to 4 segments by quartile.

HR, relative hazard ratio.

**p < 0.01, ***p < 0.001.

**FIGURE 1:**
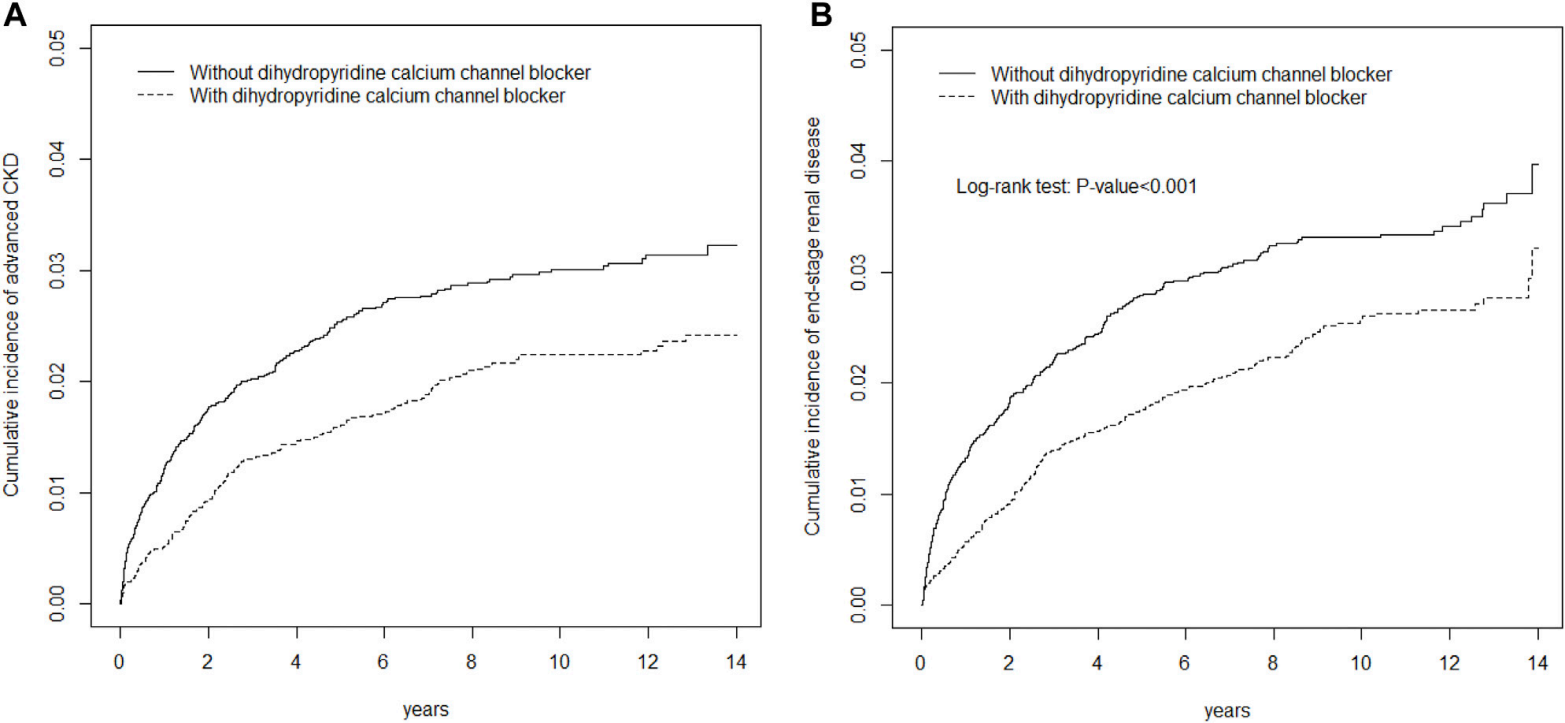
Cumulative incidence of EPO **(A)** or ESRD **(B)** curves for dihydropyridine calcium channel blocker (DCCB) users and dihydropyridine calcium channel blocker (DCCB) non-users by propensity score matched.

Figure 1 depicts cumulative incidence curves of advanced CKD or ESRD for DCCB users and DCCB nonusers by propensity score matching. The *p* values from the log-rank tests for both cohorts were <0.001, and DCCB users were more likely to have lower advanced CKD and ESRD risks than were DCCB nonusers.

## Discussion

DM patients with CKD who used DCCBs had a lower risk of progression to advanced CKD or ESRD compared with those who did not use DCCBs. We suggested several mechanisms of DCCBs that could account for this finding: improved blood pressure reduction, improved maintenance of renal perfusion, and DCCBs’ antiatherosclerotic properties. Although the optimal blood pressure target for improving renal outcomes in patients with CKD remains unclear, the evidence supports blood pressure control through antihypertensives for patients with systolic blood pressure <130 mmHg (Chang et al., 2019). The Japanese Trial to Assess Optimal Systolic Blood Pressure in Elderly Hyperintensive Patients and the Action in Diabetes and Vascular Disease: Preterax and Diamicron MR Controlled Evaluation trial revealed that improved blood pressure control could lead to reduction in the risk of doubling of creatinine levels and progression to advanced CKD compared with the absence of blood pressure control (JATOS, 2005; Heerspink et al., 2010). By contrast, other studies have found that intensive blood pressure control does not improve renal outcomes (Ruggenenti et al., 2005; Appel et al., 2010; Ku et al., 2017). These findings suggest that improved renal outcomes require both improved blood pressure control and improved renal perfusion (Palmer, 2002; Ravera et al., 2006). DCCBs have been effective in lowering systolic blood pressure to <130 mmHg in different patient populations in combination with other antihypertensives (Haller, 2008; Matsui et al., 2009; Association, 2019). DCCBs have a beneficial effect on central systolic blood pressure and arterial stiffness (Matsui et al., 2009). In the past, a concern was raised regarding the effects of DCCBs on kidney function; DCCBs may cause vasodilatation of afferent renal arterioles, increase intraglomerular pressure, and promote proteinuria (Nosadini and Tonolo, 2002). In the present study, because individual information on baseline blood pressure, proteinuria, and eGFR were unavailable, the effects of DCCB on optimal blood pressure control, proteinuria, and eGFR were unknown for both cohorts. However, through propensity matching by antihypertensive classes and comorbidities, we demonstrated that compared with nonuse, DCCB use reduced the risk of progression to advanced CKD and ESRD, consistent with previous findings (Bakris et al., 1996; JATOS, 2005). DCCBs could both improve blood pressure control and preserve renal perfusion through the vasodilatory effect of systemic arterioles and renal arterioles, thus resulting in improved renal outcomes—reduced advanced CKD and ESRD risks. Further, Orekhov et al. have found DCCB could decrease the incorporation of [3H] thymidine, lower the intracellular cholesterol level, and inhibit proliferative activity of cultured cells, which exhibited antiatherosclerotic and atherogenic ability of DCCB in their *in vitro* models (Orekhov et al., 1988). A multicenter study found that DCCBs slow the progression of plaque volume in patients with hypertension (Kojima et al., 2011). Another study revealed that DCCBs could help slow the progression of minimal atherosclerotic lesions of coronary arteries (Waters et al., 1990). Because atherosclerosis and atherogenic factors could lead to early renal injuries and promote CKD progression (Chade et al., 2005; Kalra et al., 2005), DCCBs, with their antiatherosclerotic properties, would help reduce the risk of progression from early CKD to advanced CKD and ESRD.

Notably, in the present study, the DCCB users aged ≥65 years had the lowest advanced CKD and ESRD risks, suggesting DCCB safety and effectiveness in elderly DM patients with CKD. Elderly patients, particularly those with DM and CKD, may glean more benefits from DCCB use because advanced age is associated with greater arterial stiffness (Scuteri et al., 2018; Galvão et al., 2020). Future studies investigating the renal benefits of DCCBs for elderly patients with DM are warranted. Our study also found a dose–response relationship with DCCB use: of all patients, patients with the longest duration of DCCB use (i.e., >1,100 days) had the lowest advanced CKD and ESRD risks, which further strengthens the evidence of the benefits of DCCBs on renal outcomes.

This study has several limitations. First, participant information on systolic and diastolic blood pressure, pulse pressure, glycated hemoglobin level, glucose level, body mass index, low density lipoprotein level, triglyceride level, family history of kidney disease (including immunoglobulin A nephropathy or autosomal dominant polycystic kidney disease), and exercise habits was unavailable in NHIRD. Second, information on serum creatinine, eGFR, urine albumin-to-creatinine ratio, and urine protein-to-creatinine ratio was also lacking in NHIRD. The NHI program has stated that patients whose eGFR is < 15 ml/min/1.73 m^2^ and whose hemoglobin levels is < 10 gm/dL may receive the official EPO prescription benefit package. Because anemia is common in DM patients with CKD, using EPO prescription as a proxy for advanced CKD was reasonable. Third, medication compliance and blood pressure and glucose control among the patients could not be ensured. Also, those individuals on DCCBs may be inherently different from those not on the prescription hence the observed difference rather than the drug effect as the observed difference would be potential confounding by indication. Fourth, the dosage of the antihypertensives, as well as T-type or L-type DCCBs, was not considered. Fifth, ACEIs or ARBs were not completely matched; thus, their effects might not have been eliminated completely. We have used time-dependent model in this study, thus potential immortal time bias in studies for evaluating the effect of drug would be eliminated. Sixth, residual confounding might exist, and we did not run negative control outcome analysis to assess residual confounding should be announced here. Finally, response to antihypertensive treatment involves gene polymorphisms (Stavroulakis et al., 2000; Schelleman et al., 2004). Therefore, the results of this nationwide, population-based cohort study may not be directly applicable to other racial or ethnic populations.

In conclusion, DM patients with CKD who used DCCBs had lower risk of progression to advanced CKD and ESRD compared with those who did not use DCCBs. This study demonstrated the individual renal benefits of DCCBs in DM patients with CKD. Additional randomized controlled trials to confirm these findings are required.

## Data Availability

The original contributions presented in the study are included in the article/Supplementary Material, further inquiries can be directed to the corresponding author.

## Supplementary Material

Supplementary Figure S1 Distribution of covariates before propensity-scorematching.

## Abbreviations

aHR, adjusted hazard ratio; CI, confidence interval; ICD-9-CM, International Classification of Diseases, Ninth revision, Clinical Modification; IQRs, interquartile ranges; LHID, Longitudinal Health Insurance Database; NHIRD, National Health Insurance Research Database.
