# Supplementary material for: Chronic Kidney Disease Progression Risk in Patients With Diabetes Mellitus Using Dihydropyridine Calcium Channel Blockers: A Nationwide, Population-Based, Propensity Score Matching Cohort Study
Source: Front Pharmacol. 2022 Mar 9;13:786203. doi: 10.3389/fphar.2022.786203 (PMC8959929; doi:10.3389/fphar.2022.786203)
Supplement: Supplementary file 1 [file Table1.docx]

**Supplement Table 1.**

| ICD-9 code | Disease |
| --- | --- |
| 250.x0 and 250.x2 | Type 2 diabetes |
| 585 | Chronic kidney disease |
| 585 | ESRD |
| 140-208 | Cancer |
| 272 | Hyperlipidemia |
| 430-438 | Stroke |
| 491, 492, 496 | COPD |
| 571 | Cirrhosis |
| 427 | Arrhythmia |
| 398.91, 402.01, 402.11, 402.91, and 428 | Congestive heart failure |
| 729.1 | Fibromyalgia |
| 410-414 | Coronary artery disease |
| 291, 303, 305, 571.0, 571.1, 571.2, 571.3, 790.3, and V11.3 | Alcohol-related diseases |
| 440.0, 440.2, 440.3, 440.8, 440.9, 443, 444.0, 444.22, 444.8, 444.9, 447.8, and 447.9 | PAOD |
| 592, 594 | Renal stone |
| 531-534 | Peptic Ulcer Diseasse |

ICSD與ICD-9 code對照

| ICD-9-CM(Diseases) | ICSD((Diseases)) |
| --- | --- |
| 307.40 Nonorganic Sleep Disorder, Unspecified |  |
| 307.41 Transient Disorder of Initiating or Maintaining Sleep | 307.41-0 Adjustment Sleep Disorder  307.41-1 Inadequate Sleep Hygiene |
| 307.42 Persistent Disorder of Initiating or Maintaining Sleep | 307.42-0 Psychophysiologic Insomnia  307.42-1 Sleep Choking Syndrome |
| 307.43 Transient Disorder of Initiating or Maintaining Wakefulness |  |
| 307.44 Persistent Disorder of Initiating or Maintaining Wakefulness |  |
| 307.45 Phase-Shift Disruption of 24-Hour Sleep-Wake Cycle | 307.45-0 Time Zone Change (Jet Lag) Syndrome  307.45-1 Shift Work Sleep Disorder  307.45-3 Irregular Sleep-Wake Pattern |
| 307.46 Somnambulism or Night Terrors | 307.46-0 Sleepwalking  307.46-1 Sleep Terrors  307.46-2 Confusional Arousals |
| 307.47 Other Dysfunctions of Sleep Stages or Arousal from Sleep | 307.47-0 Nightmares  307.47-1 Subwakefu lness Syndrome  307.47-2 Sleep Starts  307.47-3 Sleep Talking  307.47-4 Terrifying Hypnagogic Hallucinations |
| 307.48 Repetitive Intrusions of Sleep |  |
| 307.49 Other | 307.49-0 Short Sleeper  307.49-1 Sleep State Misperception  307.49-2 Long Sleeper  307.49-3 Subwakefulness Syndrome  307.49-4 Insufficient Sleep Syndrome |
| 780.50 Sleep Disturbance, Unspecified |  |
| 780.51 Insomnia with Sleep Apnea | 780.51-0 Central Sleep Apnea Syndrome  780.51-1 Central Alveolar Hypoventilation Syndrome |
| 780.52 Other Insomnia | 780.52-0 Hypnotic-Dependent Sleep Disorder  780.52-1 Stimulant-Dependent Sleep Disorder  780.52-2 Food Allergy Insomnia  780.52-3 Alcohol-Dependent Sleep Disorder  780.52-4 Periodic Limb Movement Disorder  780.52-5 Restless Legs Syndrome  780.52-6 Environmental Sleep Disorder  780.52-7 Idiopathic Insomnia  780.52-8 Nocturnal Eating (Drinking) Syndrome  780.52-9 Intrinsic-Extrinsic Sleep Disorder, NOS |
| 780.53 Hypersomnia with Sleep Apnea | 780.53-0 Obstructive Sleep Apnea Syndrome  780.53-2 Sleep-Related Neurogenic Tachypnea |
| 780.54 Other Hypersomnia | 780.54-2 Recurrent Hypersomnia  780.54-3 Menstrual-Associated Sleep Disorder  780.54-6 Toxin-Induced Sleep Disorder  780.54-7 Idiopathic Hypersomnia  780.54-8 Posttraumatic Hypersomnia  780.54-9 Intrinsic-Extrinsic Sleep Disorder, NOS |
| 780.55 Disruptions of 24-Hour Sleep-Wake Cycle | 780.55-0 Delayed Sleep-Phase Syndrome  780.55-1 Advanced Sleep-Phase Syndrome  780.55-2 Non-24-Hour Sleep-Wake Syndrome  780.55-9 Circadian Rhythm Sleep Disorder, NOS |
| 780.56 Dysfunctions Associated with Sleep Stages or Arousal from Sleep | 780.56-2 Sleep Paralysis  780.56-3 Impaired Sleep-Related Penile Erections  780.56-4 Sleep-Related Painful Erections  780.56-6 Sleep-Related Abnormal Swallowing Syndrome |
| 780.59 Other | 780.59-0 REM Sleep Behavior Disorder  780.59-1 Nocturnal Paroxysmal Dystonia  780.59-3 Sudden Unexplained Nocturnal Death Syndrome  780.59-4 Sleep-Related Laryngospasm  780.59-5 Benign Neonatal Sleep Myoclonus  780.59-6 Pregnancy-Associated Sleep Disorder  780.59-7 Fragmentary Myoclonus  780.59-9 Other Parasomnia, NOS |
