# Supplementary material for: Chronic Kidney Disease Progression Risk in Patients With Diabetes Mellitus Using Dihydropyridine Calcium Channel Blockers: A Nationwide, Population-Based, Propensity Score Matching Cohort Study
Source: Front Pharmacol. 2022 Mar 9;13:786203. doi: 10.3389/fphar.2022.786203 (PMC8959929; doi:10.3389/fphar.2022.786203)
Supplement: Supplementary file 2 [file Table2.docx]

| **Supplement Table 2.** Demographic characteristics and comorbidities in the propensity-score-matched cohorts with and without dihydropyridine Calcium Channel Blocker used among diabetes mellitus patients with chronic kidney disease. | | | |
| --- | --- | --- | --- |
| Variable | Dihydropyridine Calcium Channel Blocker | |  |
|  | No | Yes |  |
|  | N = 28804 | N =10481 |  |
|  | n(%) | n(%) | Standard mean difference^§^ |
| **Age, year** |  |  |  |
| ≤ 49 | 5908(20.5) | 2764(26.4) | 0.14 |
| 50-64 | 10263(35.6) | 4434(42.3) | 0.14 |
| 65+ | 12633(43.9) | 3283(31.3) | 0.26 |
| Mean ±(SD)^†^ | 61.8(13.8) | 58.2(12.5) | 0.27 |
| **Sex** |  |  |  |
| Female | 13553(47.1) | 4701(44.9) | 0.04 |
| Male | 15251(53.0) | 5780(55.2) | 0.04 |
| **Mean aDCSI score (SD)^†^** | 2.21(2.53) | 0.89(1.48) | 0.64 |
| **Duration of diabetes** |  |  |  |
| **Comorbidity** |  |  |  |
| Cancer | 4349(15.1) | 1613(15.4) | 0.008 |
| Hyperlipidemia | 18750(65.1) | 6940(66.2) | 0.02 |
| Stroke | 7626(26.5) | 2520(24.0) | 0.06 |
| COPD | 8303(28.8) | 2460(23.5) | 0.12 |
| Cirrhosis | 11010(38.2) | 3938(37.6) | 0.01 |
| Arrhythmia | 5391(18.7) | 1466(14.0) | 0.13 |
| Congestive heart failure | 6438(22.4) | 1514(14.5) | 0.21 |
| Fibromyalgia | 9346(32.5) | 3272(31.2) | 0.03 |
| Coronary artery disease | 13656(47.4) | 4005(38.2) | 0.19 |
| Alcohol-related diseases | 3291(11.4) | 1234(11.8) | 0.01 |
| PAOD | 2968(10.3) | 958(9.14) | 0.04 |
| Renal stone | 4005(13.9) | 1335(12.7) | 0.03 |
| PUD | 14280(49.6) | 4788(45.7) | 0.08 |
| **Medication** |  |  |  |
| Statin | 17409(60.4) | 6480(61.8) | 0.03 |
| ACEI or ARB | 22822(79.2) | 8934(85.2) | 0.16 |
| Loop diuretics | 19530(67.8) | 6360(60.7) | 0.15 |
| Thiazides | 19993(69.4) | 6789(64.8) | 0.10 |
| Potassium sparing diuretics | 11754(40.8) | 3242(30.9) | 0.21 |
| Non-Dihydropyridine Calcium Channel Blockers | 12412(43.1) | 3691(35.2) | 0.16 |
| Alpha-blockers | 11172(38.8) | 3210(30.6) | 0.17 |
| Beta-blockers | 21885(76.0) | 7407(70.7) | 0.12 |
| Insulins | 19736(68.5) | 7536(71.9) | 0.07 |
| Sulfonylureas | 23859(82.8) | 9440(90.1) | 0.21 |
| Biguanides | 25019(86.9) | 9640(92.0) | 0.17 |
| Miglitol | 523(1.82) | 256(2.44) | 0.04 |
| Acarbose | 9602(33.3) | 3997(38.1) | 0.10 |
| Thiazolidinedione | 8268(28.7) | 3786(36.1) | 0.16 |
| Dipeptidyl peptidase-4 | 24214(84.1) | 9478(90.4) | 0.19 |
| Other antidiabetic | 13894(48.2) | 5585(53.3) | 0.10 |

^§^A standardized mean difference of ≤0.1 indicates a negligible difference between the two cohorts.

CAD: coronary artery disease

COPD: chronic obstructive pulmonary disease
